# A brief international screening tool for traumatic birth and childbirth-related PTSD: the city BiTS-short form

**DOI:** 10.1136/bmjgh-2025-019216

**Published:** 2025-08-17

**Authors:** Susan Ayers, Dan Brooks Wright, Rafael A Caparros-Gonzalez, Giulia Ciuffo, Georgina Constantinou, Pelin Dikmen-Yildiz, Susan Garthus-Niegel, Hanna Grundström, Jonathan Handelzalts, Antje Horsch, Chiara Ionio, Sandra Nakić Radoš, Flávia L Osório, Valentine Rattaz, Olga Riklikiene, Lara Seefeld, Valgerður Lísa Sigurðardóttir, Rebecca Webb, INTERSECT Consortium, Stephanie Alves

**Affiliations:** 1 Centre for Maternal and Child Health Research, City St George’s University of London, London, UK; 2 College of Education, University of Nevada, Las Vegas, Nevada, USA; 3 Faculty of Health Sciences, Department of Nursing. University of Granada, and Instituto de Investigacion Biosanitaria ibs. GRANADA, Granada, Spain; 4 CRIDee, Trauma Research Unit, Department of Psychology, Catholic University of Milan, Milan, Italy; 5 Department of Psychology, Kirklareli University, Kirklareli, Turkey; 6 Institute and Policlinic of Occupational and Social Medicine, Faculty of Medicine, University of Technology Dresden, Dresden, Germany; 7 Institute for Systems Medicine (ISM), Faculty of Medicine, Medical School Hamburg, MSH, Hamburg, Germany; 8 Department of Health, Medicine and Caring Sciences, Linköping University, Linköping University, Linköping, Sweden; 9 Department of Obstetrics and Gynecology in Norrköping, and Department of Biomedical and Clinical Sciences, Linköping University, Linköping University, Linköping, Sweden; 10 School of Behavioral Sciences, Academic College of Tel Aviv-Yafo, Tel Aviv-Yafo, Israel; 11 Institute of Higher Education and Research in Healthcare Sciences, University of Lausanne, Lausanne, Switzerland; 12 Department Woman-mother-child, Lausanne University Hospital, Lausanne, Switzerland; 13 University Department of Psychology, Catholic University of Croatia, Zagreb, Croatia; 14 Department of Neurosciences and Behavioral Sciences, Faculty of Medicine of Ribeirão Preto, University of Sao Paulo, Sao Paulo, Brazil; 15 Lithuanian University of Health Sciences, Kaunas, Lithuania; 16 Faculty of Nursing and Midwifery, University of Iceland, Reykjavík, Iceland; 17 National University Hospital, Reykjavík, Iceland

**Keywords:** Global Health, Maternal health, Obstetrics, Public Health, Screening

## Abstract

**Introduction:**

Screening to identify traumatic births and childbirth-related post-traumatic stress disorder (CB-PTSD) is critical for reducing the global burden of maternal mental health challenges. Despite this, no brief, validated tools exist for international use. This study therefore developed and validated a short version of the City Birth Trauma Scale (City BiTS) to provide a brief, globally relevant screening tool.

**Methods:**

The City BiTS-Short was developed in three stages. In stage 1, exclusive lasso statistical analyses were conducted on survey data of 11 302 postpartum women in 31 countries to identify the most effective items for the City BiTS-Short, ensuring all four CB-PTSD symptom domains were represented. In stage 2, stakeholder reviews were conducted with researchers, health professionals (midwives, health visitors, psychiatrist, psychologist) and representatives of women who experienced traumatic birth. In stage 3, the City BiTS-Short was finalised and psychometric properties examined across diverse geographical settings.

**Results:**

The City BiTS-Short comprises one item assessing traumatic birth and four items assessing CB-PTSD symptoms: re-experiencing, avoidance, negative cognitions and mood and hyperarousal. The scale had strong psychometric properties, including good internal consistency (α=0.78) and high correlations with the original City BiTS (r=0.90), birth trauma ratings (r=0.50), distress (r=0.56), impairment (r=0.47) and CB-PTSD diagnoses (r=0.54). It identified 90% of participants with a CB-PTSD diagnosis. Women who had operative births (F(3,2174)=127.38, p<0.001), maternal complications (F(2,2163)=212.84, p<0.001), infant complications (F(2,1100)=138.93, p<0.001) or depression (t(3209.5)=−30.96, p<0.001) had higher scores. Psychometric properties were consistent across most international contexts, with stakeholders affirming its utility.

**Conclusion:**

The City BiTS-Short offers a brief, validated screening tool for identifying birth trauma and CB-PTSD symptoms. Its widespread adoption can enhance early detection and support for women, potentially reducing the global burden of birth trauma and improving maternal mental health outcomes worldwide. Further research is needed to explore its use in specific contexts.

**WHAT IS ALREADY KNOWN ON THIS TOPIC**

- Childbirth can result in psychological trauma and childbirth-related post-traumatic stress disorder (CB-PTSD), which affects 1–36% of women globally.
- Early identification and intervention for CB-PTSD are critical, yet no consensus on screening methods currently exists, as emphasised by recent government inquiries into birth trauma.

**WHAT THIS STUDY ADDS**

- This study developed and validated a brief screening tool (the City Birth Trauma Scale (City BiTS)-Short) with robust psychometric properties and cross-cultural reliability in 31 countries.
- It provides an internationally applicable tool to identify women and their partners who experience birth trauma and CB-PTSD symptoms, enabling timely support.

**HOW THIS STUDY MIGHT AFFECT RESEARCH, PRACTICE OR POLICY**

- The City BiTS-Short marks a significant advance in the early identification of traumatic birth and CB-PTSD symptoms worldwide, with a potential role in policy and practice to reduce the global burden of traumatic childbirth and its impacts.

## Introduction

 Childbirth can be a transformative experience, but for some women, it becomes a source of psychological trauma, which can lead to childbirth-related post-traumatic stress disorder (CB-PTSD).^1^ Worldwide, between 1% and 36% of women develop CB-PTSD,^2^ with meta-analytic estimates of 4% overall.^3^ An additional 12% of women experience symptoms that cause considerable distress, despite not meeting diagnostic criteria.^3^ Early identification and intervention for those affected are critical, as emphasised in international recommendations aimed at preventing traumatic births and reducing CB-PTSD.^4^,^6^ Similarly, a recent government inquiry into birth trauma in the UK stressed the urgent need for the development of a brief, valid and effective screening tool to address this gap.^5^

The City Birth Trauma Scale (City BiTS)^7^ offers one solution. A recent systematic review of measures of psychological birth trauma recommended the City BiTS as a credible tool for assessing birth trauma in clinical practice.^8^ This 29-item questionnaire measures CB-PTSD symptoms according to psychiatric diagnostic criteria, encompassing exposure to traumatic stressor(s), symptoms of re-experiencing, avoidance, negative cognitions and mood, hyperarousal, symptom duration, distress, impairment and exclusion of other causes.^9^ Versions of the City BiTS are also available for fathers or birth partners^10^ and maternity staff.^11^ It has been used internationally across diverse populations with consistent psychometric properties,^12^,^21^ making it a robust tool for worldwide use. Symptom scores are strongly associated with distress and impaired functioning,^7^ highlighting the scale’s relevance for identifying those in need of support.

A potential barrier to using the City BiTS in healthcare settings is that it has 29 items, so takes time to complete. This may prevent its use as a screening tool in healthcare services, particularly in less resourced settings. This study therefore aimed to develop a short form of the City BiTS—the City BiTS-Short—which can be used to screen for birth trauma and CB-PTSD symptoms to identify those who need more detailed assessment and support, therein advancing efforts to mitigate the psychological impact of traumatic births and improve global maternal health.

## Methods

### Design

Development and validation of the City BiTS-Short was conducted in three stages. In the first stage, data from the INTERSECT project^22^ (dataset V.1, 2024)^23^ was used to statistically identify the best items to create a short form between four and 10 items long, ensuring items from all four PTSD symptom clusters were included. Stage 2 involved consultation and review by three groups of stakeholders: (1) international researchers who had translated and validated the City BiTS into another language; (2) members of an organisation representing women with experience of birth trauma and (3) health professionals. In the final stage, data from the INTERSECT project were used to determine the psychometric properties of the City BiTS-Short.

### Participants

INTERSECT 2024 includes data for 11 302 women at 6–12 weeks post partum (M=8.5, SD=1.9 weeks) from 31 countries (Australia, Brazil, Chile, Croatia, Cyprus, Czechia, Estonia, Germany, Iceland, Ireland, Israel, Italy, Lithuania, Malawi, Nepal, Nigeria, Norway, Pakistan, Poland, Portugal, Romania, Saudi Arabia, Serbia, Slovakia, Slovenia, Spain, Sweden, Switzerland, Türkiye, United Arab Emirates and UK). Data were collected from 2021 to 2024. Inclusion criteria were that participants: (1) gave birth to a baby in the previous 6–12 weeks; (2) were legally adults in the country they resided in (ie, aged 16 years or 18 years or over) and (3) gave their informed written or verbal consent to participate in INTERSECT and for anonymised data to be part of the international dataset. Anonymised data were shared securely, harmonised and linked to create the INTERSECT 2024 dataset.^22 23^

Three stakeholder groups with a total of 24 participants provided feedback on the City BiTS-Short: (1) researchers who had translated and validated the City BiTS in another language (Croatian, French, German, Hebrew, Icelandic, Italian, Lithuanian, Portuguese, Spanish, Swedish and Turkish) (n=12); (2) members of a UK organisation representing those with lived experience of birth trauma (n=3), one of whom was a perinatal psychiatrist; (3) health professionals with experience working with women in maternity or postpartum care in Iceland, Ireland, Sweden or the UK (n=9: four midwives, four health visitors, one psychologist). Most participants were women (n=22, 92%).

### Patient and public involvement

Patients and public were involved in the development of the City BiTS-Short from inception. Discussions with Patient and public involvement (PPI) representatives informed how the scale was developed in terms of stakeholder reviews and feedback (see the Procedure section), as well as the content of the City BiTS-Short (see the Results section). PPI representatives canvassed views on the draft versions of the City BiTS-Short from women with lived experience. PPI representatives will disseminate the City BiTS-Short via their organisation and to the wider public.

### Measures

The following measures were analysed to develop the City BiTS-Short:

The City BiTS^7^ has 29 items that measure PTSD symptoms according to Diagnostic and Statistical Manual of Mental Disorders-5(DSM-5) diagnostic criteria,^9^ but where symptoms are specifically related to events of labour and birth or immediately before or after the birth. The scale measures stressor criterion (two items) and PTSD symptoms of re-experiencing (five items), avoidance (two items), negative cognitions and mood (six items), hyperarousal (seven items) and dissociation (two items). Symptoms are rated for frequency over the last week from 0 (*not at all*), 1 (*once*), 2 (*2–4 times*) or 3 (*5 or more times*). Total scores are calculated by combining items from the re-experiencing, avoidance, negative cognitions and mood and hyperarousal subscales, with a possible range of 0–60. The scale has good reliability and psychometric characteristics across translations.^12^,^21^ Psychometric studies suggest the scale has two factors: (1) birth-related symptoms (re-experiencing and avoidance symptoms) and (2) general symptoms (negative cognitions and mood, hyperarousal).^712^,^21^ Internal consistency in the INTERSECT sample was high (Cronbach’s ɑ=0.93).

The Edinburgh Postnatal Depression Scale^24^ was used to measure symptoms of depression. This is a 10-item scale measuring symptoms of depression on a response scale from 0 to 3 (range 0–30). A cut-off of 13 or more was used to classify those with probable depression.^25^

Childbirth variables included self-reported information on whether birth was traumatic (0 *not at all traumatic–*10 *extremely traumatic*), type of birth (vaginal, assisted vaginal, emergency caesarean, elective caesarean) and maternal or infant complications during birth (major, minor, none).

### Procedure

Approval was obtained via the UK Data Service to conduct secondary analyses on the INTERSECT 2024 dataset^23^ to develop and test the City BiTS-Short. Details of the methods and sample of the INTERSECT study are reported elsewhere.^2^ The survey was conducted according to a standard protocol.^22^ The INTERSECT survey questions and measures were consistent across countries and translated using standard cultural adaptation procedures.^26^ Participants were recruited through universal health or family services attended by pregnant or postpartum women, for example, hospitals, clinics, birth centres. Surveys were completed between 6 weeks and 12 weeks post partum and were completed online, by pen and paper or telephone interview according to each location’s conditions for recruitment and participation.

Development and testing of the City BiTS-Short was conducted in three stages:

#### Stage 1

The exclusive Lasso (Least Absolute Shrinkage and Selection Operator) statistical technique^27^ was used to reduce the number of items assessing symptoms (criteria B–E) in the City BiTS. Exclusive lasso is a statistical technique used in data analysis and machine learning to select important variables and prevent overfitting in a model. It involves variable selection and shrinkage to simplify a model in a way that maximises fit to the data. The exclusive lasso was constrained using preset criteria to create the City BiTS-Short as follows:

1. That the City BiTS-Short is brief in order to provide a quick screening tool. The exclusive lasso was therefore limited to produce results with between four and 10 items.
2. That items in the City BiTS-Short must cover the four PTSD symptom components of re-experiencing (B), avoidance (C), negative cognitions and mood (D) and hyperarousal (E), with at least one item for each component.
3. The international researchers consulted did not report any of the items to be problematic.
4. That the scoring of the City BiTS-Short is simple.
5. That scores on the City BiTS-Short and City BiTS are highly correlated; and that this should hold true for the scale in all countries for which we had data.

#### Stage 2

Consultations were conducted with three groups of stakeholders to ensure relevance, acceptability and feasibility for postpartum women, researchers and health professionals. To ensure international relevance and validity, researchers from nine countries were emailed to ask if any of the items on the original scale were less optimal in translations and validations of the City BiTS. Responses to this informed decisions about which version of the City BiTS-Short to take forward to subsequent consultations. To ensure acceptability and relevance to women who experience a traumatic birth, possible versions of the City BiTS-Short were reviewed by members of an organisation for women with lived experience of birth trauma in the UK. Finally, to ensure clinical acceptability and feasibility, versions of the City BiTS-Short were reviewed by health professionals from the UK, Iceland, Ireland and Sweden. For these consultations, draft versions of the City BiTS-Short were sent to stakeholders before conducting four online focus groups (n=3, n=5, n=2, n=2) to obtain their views and feedback. Notes were made during the meetings to record and summarise feedback.

#### Stage 3

Results of stages 1 and 2 were used to finalise the City BiTS-Short and conduct further statistics to ensure good fit to the data and good psychometric properties of the City BiTS-Short when used across countries and within different countries.

### Analysis

To find a good subset of items, we used the ExclusiveLasso package from R.^28^ The exclusive lasso is a technique used to choose a subset of items in a multiple regression context.^27^ It works by constraining the size of the sum of the absolute values of the standardised coefficients (ie, the *β* values).^27^ The exclusive lasso was run twice—first on the City BiTS 20 items assessing CB-PTSD symptom components (criteria B, C, D and E) and then on the 10 items assessing CB-PTSD symptoms that remained after removing items highlighted by stakeholders as problematic. The exclusive lasso forced at least one item from each designated component to be included in the final set of items. To evaluate whether the items fitted well for all countries, the sum of the resulting subset was compared with the sum of the long scale using Pearson’s correlation, and the internal consistency of the items was examined with Cronbach’s α. A technical report with more details on the analyses is available in online supplemental material. Substantive analyses were conducted with R statistical software V.4.4.1.^29^

## Results

### Reducing the number of items

Researchers recommended the following items should be removed. Words like *‘flashbacks*’ and *‘jumpy*’ were difficult to translate into some languages, so items using these words were removed. Other items were removed because they had less optimal psychometric characteristics in some countries, for example, less variance, less optimal factor loadings or less relevance to postpartum women.

B2: *bad dreams or nightmares about the birth (or related to the birth*).

B3: *flashbacks to the birth and/or reliving the experience.*

C2: *trying to avoid things that remind me of the birth (eg, people, places, TV programmes*).

D1: *not able to remember details of the birth.*

D4: *feeling negative about myself or thinking something awful will happen.*

E1: *feeling irritable or aggressive.*

E4: *feeling jumpy or easily startled.*

E5: *problems concentrating.*

E6: *not sleeping well because of things that are not due to the baby’s sleep pattern.*

In addition, representatives of women with lived experience suggested that part of item B5 *‘Feeling tense or anxious when reminded of the birth*’ overlapped with item E3 *‘Feeling tense and on edge*’, and that other B items might be more representative of re-experiencing symptoms. Item B5 was therefore removed. This left 10 items assessing symptoms B–E.

#### Exclusive lasso

**Figure 1:**
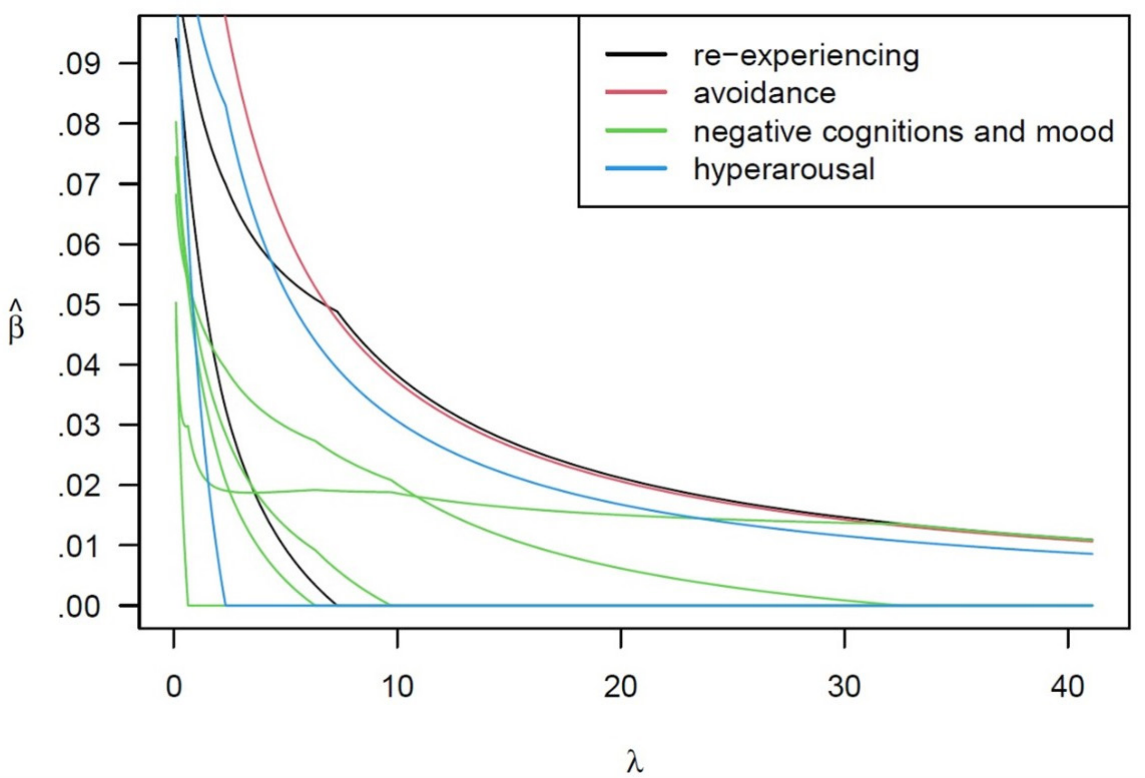
Results of the exclusive lasso.

Results of the exclusive lasso are shown in figure 1. As the shrinkage in the λ parameter increased, the estimates for the *β*s tended to decrease. Only four items—one from each component—remained above zero at the λ parameter of 40 and were of a similar size.

Each possible combination for the City BiTS-Short was correlated with the City BiTS and the values for Cronbach’s α examined (see online supplemental table 1). Both these measures increased with more items, which is to be expected. However, the four-item City BiTS-Short correlated well with the total City BiTS (*r*=0.90) and showed good internal consistency (α=0.78).

### Refining the City BiTS-Short

Consultation and review by health professionals and women with lived experience resulted in further refinements:

1. *Brevity:* there was a strong preference for as few items as possible, and the importance of each symptom component being equally represented was raised. The four-item City BiTS-Short symptom scale was therefore ideal, with those items remaining above zero on the far right of figure 1 retained. These assess symptoms B–E.
2. *Stressor:* the importance of retaining a question about birth as a traumatic stressor (DSM-5 criterion A) was discussed and agreed. However, representatives of women with lived experience thought the criterion A items in the City BiTS of whether women thought they or their baby might be seriously injured or die ‘might be silencing’ for women who have traumatic births without threat of serious injury or death. The importance of criterion A has also been questioned in the diagnostic nomenclature where it is argued that the presence of symptoms B–E should be sufficient.^30^ As the purpose of the City BiTS-Short is to screen not to diagnose, criterion A questions were replaced with one question of *‘Did you find any part of the birth distressing or traumatic?’* (Yes/No). This ensures that those who experience trauma in the absence of threat of injury or death are identified. The wording of this question was provided by representatives of women with lived experience and health professionals who already use it in their work.
3. *Instructions:* on the City BiTS, the instructions are split across an introductory paragraph and a footnote. In the City BiTS-Short, these instructions were combined so it is clear that if the traumatic event occurred before or after birth (eg, the baby needing intensive care), the scale should be completed in relation to this event. Instructions were also revised to make the language more inclusive so the scale can be used with fathers and birth partners as well as women.
4. *Baby’s date of birth:* the date the baby was born is often available to health professionals but not to researchers. Retaining this field enables participants’ responses to be matched to medical or research records. It was therefore retained but moved to the header so it can easily be omitted if not required.
5. *Non-symptom items:* there were mixed views on whether the City BiTS-Short should include one or all of the non-symptom items. These items assess: when symptoms started, the duration of symptoms (criterion F), distress and impaired functioning (criterion G) and possible differential diagnosis (criterion H). Stakeholders identified none, one or two of these questions as important to retain, with very little overlap or consensus. Different views reflected the purpose and context in which respondents would be using the scale. Thus, to ensure the City BiTS-Short is brief and relevant to as many contexts as possible, it was decided to omit these items, with the caveat that people using the City BiTS-Short can choose to include some or all of the non-symptom items if it is relevant to their context and purpose.

Overall, health professionals and representatives of women with lived experience thought the City BiTS-Short provided a valuable tool with which to start a conversation about birth trauma and identify women who require further assessment and referral. The final City BiTS-Short was thought to be a ‘safe conversation starter’ which could ‘help direct that initial conversation’ (health professionals). It was also noted that items in the City BiTS-Short cover both the four PTSD symptom components and many of the postpartum emotional responses health professionals see in their work (health professionals). The City BiTS-Short was thought to be ‘extremely useful’ for research, especially studies with multiple measures where it is important to minimise the burden on participants (researchers).

### The City BiTS-Short

Items of the final City BiTS-Short are shown in table 1 and the complete City BiTS-Short is given in the online supplemental appendix.

**Table 1:** The City BiTS-Short

Item	PTSD component	Wording	Response scale
A	Stressor	Did you find any part of the birth distressing or traumatic?	Yes/No
B4	Re-experiencing	Getting upset when reminded of the birth	0–3
C1	Avoidance	Trying to avoid thinking about the birth	0–3
D3	Negative cognitions and mood	Feeling strong negative emotions about the birth (eg, fear, anger, shame)	0–3
E3	Hyperarousal	Feeling tense or on edge	0–3

**Figure 2:**
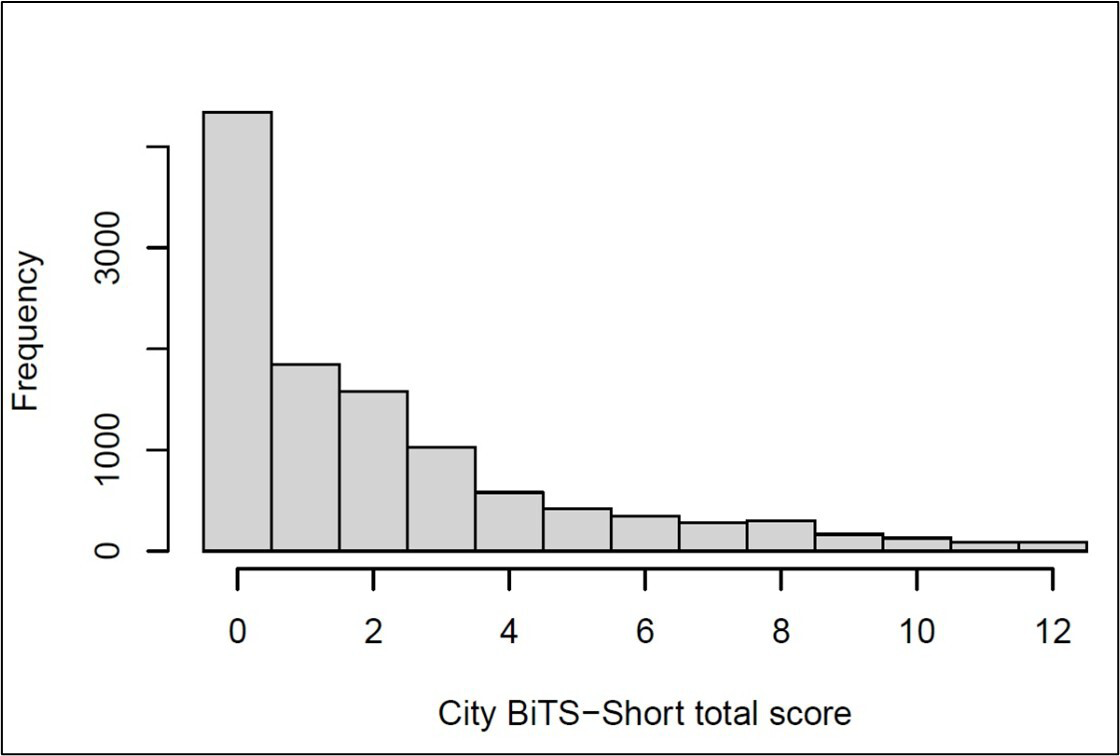
Histogram of City Birth Trauma Scale (City BiTS)-Short scores.

The first item, *‘Did you find any part of the birth distressing or traumatic?’* is rated yes/no (1/0). CB-PTSD symptoms (B–E) are reported for the *last week* and items are scored in the same way as the City BiTS: 0=*not at all*, 1=*once*, 2=*2–4 times* and 3=*5 or more times*. Total symptom scores for the City BiTS-Short range from 0 to 12. Distribution of City BiTS-Short scores is shown in figure 2 and shows that a large group of women (39%) did not report any symptoms (ie, scored 0), most of whom probably did not have a traumatic birth. Thus, it is reasonable to suggest that when the City BiTS-Short is used as a screening tool, if women respond ‘no’ to the first question, they do not need to complete the rest of the items.

Respondents who reply ‘yes’ to the first item and report symptoms on items B–E should be followed up for fuller assessment, support or treatment as appropriate. Examination of the ability of scores on the City BiTS-Short to predict a CB-PTSD diagnosis using a logistic regression showed a score of 7 identified approximately 25% of those with diagnostic CB-PTSD, and a score of 12 identified approximately 90% of those with diagnostic PTSD (see online supplemental figure 3).

#### Validity

The City BiTS-Short had good internal consistency (Cronbach α 0.78). Correlations between items on the City BiTS-Short and distress, impairment, the total City BiTS and a PTSD diagnosis are shown in table 2. This shows that the four symptom items correlate between *r*=0.32 and *r*=0.65, with lowest correlations for the hyperarousal item (E3). Construct validity was supported by the strong correlation between the City BiTS-Short and the City BiTS (*r*=0.90). Convergent validity was confirmed by City BiTS-Short being correlated with ratings of birth as traumatic (*r*=0.50, p<0.001), symptoms causing distress (*r*=0.56, p<0.001) and impairment (*r*=0.47, p<0.001) and with CB-PTSD diagnosis (*r*=0.54, p<0.001). The correlation between the City BiTS-Short and CB-PTSD diagnosis was equivalent to that observed between the longer City BiTS and CB-PTSD diagnosis (*r*=0.55, p<0.001).

**Table 2:** Correlations between items on the City BiTS-Short, distress, impairment and total City BiTS scores

B4 Re-experiencing	C1 Avoidance	D3 NCAM	E3 Hyperarousal	Distress	Impairment	City BiTS	City BiTS-Short
C1 Avoidance	0.65
D3 NCAM	0.64	0.62
E3 hyperarousal	0.32	0.32	0.37
Distress	0.36	0.37	0.44	0.52
Impairment	0.30	0.31	0.37	0.45	0.67
City BiTS	0.81	0.81	0.83	0.68	0.56	0.47
City BiTS-Short	0.70	0.69	0.72	0.69	0.63	0.57	0.90
PTSD diagnosis	0.43	0.51	0.49	0.31	0.38	0.34	0.55	0.54

* All correlations were significant at p<0.001.

City-BiTS, City Birth Trauma Scale; NCAM, negative cognitions and mood; PTSD, post-traumatic stress disorder.

Criterion validity was examined through known groups analysis. This confirmed that CB-PTSD symptoms as measured by the City BiTS-Short were greater in women who had major (*M*=4.11) or minor complications (*M*=2.79) during birth, compared with those with no complications (*M*=1.89) (*F*(2 2163)=212.84, p<0.001). Similarly, City BiTS-Short CB-PTSD symptoms were greater in women who had an emergency caesarean (*M*=3.30), followed by assisted vaginal birth (*M*=2.86), elective caesarean (*M*=2.06), and unassisted vaginal birth (*M*=1.78) (*F*(3 2174)=127.38, p<0.001). Women whose infants had major birth complications also had more City BiTS-Short CB-PTSD symptoms (*M*=4.11) compared with those whose infants had minor complications (*M*=2.79) or no complications (*M*=1.89) (*F*(2 1100)=138.93, p<0.001). Finally, women with probable depression had greater City BiTS-Short CB-PTSD symptoms (*M*=3.85) compared with women without depression (*M*=1.61) (*t*(3209.5)=−30.96, p<0.001).

#### Psychometric properties across countries

One of the criteria for choosing items for the City BiTS-Short is that they behave similarly with samples in different countries. Table 3 shows information on the distribution of symptoms (range, mean, SD), internal consistency (Cronbach α), convergent validity (correlation with ratings of birth as traumatic) and construct validity (correlations between the City BiTS-Short and City BiTS) for 31 countries. Internal consistency (Cronbach α) ranged from 0.53 (Türkiye) to 0.95 (Serbia), with most being around 0.70. As the size of α is closely related to the number of items, these values are high considering there are only four items in the City BiTS-Short and these items were chosen specifically to measure different components of CB-PTSD.

**Table 3:** Internal consistency, construct and convergent validity in different countries

Country	Total N	Symptoms Mean (SD)	Internal consistency		Correlation with the City BiTS		Correlation with birth trauma	
		Range	α	95% CI	R	95% CI	R	95% CI
Australia	166	2.87 (2.69) 0–11	0.64	0.55 to 0.73	0.88	0.84 to 0.91	0.52	0.40 to 0.62
Brazil	596	1.75 (2.67) 0–12	0.75	0.72 to 0.78	0.86	0.84 to 0.88	0.48	0.42 to 0.54
Chile	127	1.83 (2.33) 0–10	0.69	0.59 to 0.77	0.86	0.81 to 0.90	0.41	0.25 to 0.55
Croatia	380	1.59 (2.18) 0–12	0.68	0.62 to 0.73	0.84	0.81 to 0.87	0.54	0.46 to 0.60
Cyprus	142	2.01 (2.12) 0–10	0.64	0.53 to 0.73	0.86	0.80 to 0.89	0.52	0.38 to 0.63
Czechia	246	1.64 (2.19) 0–12	0.70	0.64 to 0.76	0.87	0.83 to 0.90	0.45	0.34 to 0.54
Estonia	285	2.27 (2.57) 0–11	0.68	0.62 to 0.74	0.86	0.83 to 0.89	0.48	0.39 to 0.57
Germany	1644	1.47 (1.80) 0–11	0.62	0.59 to 0.65	0.84	0.82 to 0.85	0.43	0.39 to 0.47
Iceland	701	1.43 (2.18) 0–12	0.69	0.65 to 0.72	0.88	0.86 to 0.89	0.54	0.48 to 0.59
Ireland	274	1.88 (2.52) 0–12	0.76	0.71 to 0.80	0.89	0.86 to 0.91	0.60	0.52 to 0.67
Israel	248	1.42 (2.18) 0–12	0.73	0.67 to 0.78	0.89	0.86 to 0.91	0.46	0.36 to 0.55
Italy	211	2.04 (2.45) 0–12	0.70	0.62 to 0.76	0.84	0.79 to 0.88	0.53	0.42 to 0.62
Lithuania	328	1.42 (2.04) 0–10	0.67	0.60 to 0.72	0.86	0.83 to 0.88	0.54	0.45 to 0.61
Malawi	248	4.47 (3.73) 0–12	0.82	0.78 to 0.85	0.95	0.94 to 0.96	0.79	0.74 to 0.83
Nepal	490	2.57 (3.46) 0–12	0.87	0.85 to 0.89	0.95	0.94 to 0.96	0.27	0.19 to 0.35
Nigeria	406	0.33 (0.98) 0–8	0.71	0.66 to 0.75	0.86	0.83 to 0.88	0.25	0.16 to 0.34
Norway	221	3.28 (3.08) 0–12	0.69	0.61 – 0.76	0.85	0.80 to 0.89	0.67	0.59 to 0.74
Pakistan	335	5.35 (3.20) 0–12	0.76	0.71 to 0.81	0.88	0.85 to 0.91	0.35	0.26 to 0.44
Poland	296	1.67 (2.45) 0–12	0.86	0.83 to 0.88	0.94	0.93 to 0.95	0.59	0.51 to 0.66
Portugal	227	1.71 (2.21) 0–12	0.77	0.72 to 0.81	0.85	0.82 to 0.88	0.52	0.41 to 0.61
Romania	135	1.69 (2.22) 0–11	0.68	0.60 to 0.74	0.84	0.80 to 0.88	0.46	0.32 to 0.59
Saudi Arabia	248	4.02 (3.28) 0–12	0.69	0.60 to 0.77	0.83	0.76 to 0.87	0.70	0.63 to 0.76
Serbia	267	1.09 (2.17) 0–11	0.95	0.94 to 0.96	0.97	0.96 to 0.98	0.46	0.36 to 0.55
Slovakia	437	2.28 (2.65) 0–12	0.76	0.70 to 0.80	0.87	0.84 to 0.90	0.58	0.52 to 0.64
Slovenia	236	1.76 (2.38) 0–12	0.77	0.75 to 0.79	0.90	0.89 to 0.91	0.58	0.48 to 0.66
Spain	254	1.57 (2.01) 0–10	0.70	0.65 to 0.74	0.89	0.87 to 0.91	0.41	0.30 to 0.51
Sweden	469	1.57 (2.37) 0–12	0.76	0.71 to 0.81	0.89	0.85 to 0.91	0.58	0.51 to 0.64
Switzerland	247	2.04 (1.84) 0–11	0.67	0.60 to 0.73	0.86	0.83 to 0.89	0.47	0.37 to 0.57
Türkiye	1013	3.69 (3.14) 0–12	0.53	to	0.83	0.79 to 0.87	0.68	0.64 to 0.71
United Arab Emirates	165	1.79 (2.08) 0–10	0.74	0.70 to 0.78	0.87	0.84 to 0.89	0.13	−0.02 to 0.28
United Kingdom	260	2.24 (2.94) 0–12	0.79	0.74 to 0.83	0.90	0.87 to 0.92	0.62	0.54 to 0.69

City BiTS, City Birth Trauma Scale.

Construct validity (correlations between total scores on the City BiTS-Short and City BiTS) ranged from 0.83 (Saudi Arabia, Türkiye) to 0.97 (Serbia), and so, it was high for a screening instrument. Convergent validity (correlation with ratings of birth as traumatic) had a wider range from 0.13 (United Arab Emirates) to 0.79 (Malawi), with most correlations being over 0.40.

## Discussion

This study successfully developed a short version of the City BiTS to screen for birth trauma and CB-PTSD symptoms. The City BiTS-Short has one item assessing whether respondents had a traumatic birth, and four items assessing PTSD symptoms of re-experiencing, avoidance, negative cognitions and mood and hyperarousal. The City BiTS-Short has good psychometric properties, including internal consistency, convergent and criterion validity. These properties were consistent across most international settings, although additional research is needed to explore the convergent validity of the tool in certain contexts. The City BiTS-Short was positively evaluated by health professionals, researchers and representatives of women with lived experience, further supporting its utility.

The City BiTS-Short therefore provides a promising, brief screening tool to identify women or birth partners who had traumatic birth experiences and have symptoms of CB-PTSD. It can be administered by self-report questionnaire or by health professionals or researchers, and there is the option to add some or all of the non-symptom items (F–H) where relevant. However, the City BiTS-Short is not a diagnostic tool, so those who report symptoms should be followed up with a fuller assessment using the City BiTS or other means. Further research is needed to examine the diagnostic accuracy of the City BiTS-Short and whether different ways of administering the scale or the inclusion of non-symptom items affect its reliability and validity.

### Implications for clinical practice

Research clearly shows the importance of asking about birth experiences and birth trauma. Studies show that women want health professionals to recognise their need to discuss and process their birth experiences.^31^ Reviews of CB-PTSD and uptake of treatment show that women want health professionals to ask about their birth and possible trauma in a non-judgmental way at different time points and provide support.^32^

The City BiTS-Short can help achieve this. It was judged to be a ‘safe conversation starter’ by health professionals, it takes very little time to complete and provides immediate results. If a person responds ‘no’ to the first question on whether the birth was distressing or traumatic, there is no need for them to complete the rest of the questions. Thus, it can be used with women or their partners after birth to start a conversation about their birth experiences and identify those who might need additional support in a quick and efficient way. If women or their partners respond ‘yes’ to having a traumatic birth and report CB-PTSD symptoms, this can be followed up either at the time or through referral to specialist services.

The question of appropriate criteria for further assessment or referral requires further evaluation. Our findings suggest the highest scores on the City BiTS-Short identify 90% of women with a CB-PTSD diagnosis. However, given the evidence that women with subdiagnostic symptoms report substantial distress and impairment,^2 3^ we believe a more inclusive approach to screening and assessment is preferable. We therefore recommend the City BiTS-Short is used as part of a two-stage screening process, where those who report a traumatic birth and one or more CB-PTSD symptoms undergo further assessment using the City BiTS or clinical evaluation. Routine evaluation of screening programmes is critical to determine the effectiveness of different criteria and refine the implementation and use of the City BiTS-Short.

Furthermore, the implementation and effectiveness of screening programmes depends substantially on contextual factors, including healthcare policies and practice in each country and healthcare organisation, how the questionnaire is administered (eg, verbal, digital) and who is responsible for administering the screening tool.^33^,^35^ Moreover, the lack of consensus on whether to include items about non-symptom diagnostic criteria (eg, distress and impairment) shows how the usefulness of these items differs depending on the specific context in which it is used. Given this, the context of screening is an important consideration. Services may opt to include non-symptom questions alongside the City BiTS-Short if these questions are deemed contextually relevant. Training health professionals to ensure they take a standardised and effective approach within that context is essential.

### Implications for research

For research purposes, the City BiTS-Short has the advantage of being a reliable and valid brief measure of traumatic birth and core CB-PTSD symptoms, with the option to include non-symptom items if relevant. This is particularly advantageous for research involving multiple questionnaire measures, where CB-PTSD is not the primary outcome of interest and/or where participant burden needs to be minimised. Another advantage is that the City BiTS is already translated into over 30 languages, so the City BiTS-Short will be available in different languages quite quickly. The disadvantage is that the City BiTS-Short does not provide a measure of diagnostic PTSD, although diagnostic criteria are subject to debate^30^ and change.^9^

The City BiTS-Short requires further investigation in several key areas. These include assessing its reliability and validity across different administration methods (eg, questionnaire, inperson, online), both with and without the optional non-symptom items. Further psychometric tests are needed, including examining the scale’s cultural invariance. Finally, as mentioned above, it is essential that the effectiveness of the City BiTS-Short when it is integrated into clinical pathways for identifying and treating CB-PTSD is evaluated. This includes evaluating implementation factors, such as the uptake and acceptability of routine screening using the City BiTS to women and health professionals and country-specific barriers and facilitators to implementation and uptake.

### Strengths and limitations

This study represents the first effort to develop and validate a short screening tool for CB-PTSD, founded on the validated City BiTS and data from 31 diverse countries. As a rigorously developed and robust measure, the City BiTS-Short aligns with international objectives to enhance maternal health and obstetric care.^36^,^38^ However, certain limitations need to be considered. The City BiTS-Short was developed and validated within the same dataset, encompassing participants from multiple countries and ethnicities. While this supports generalisability across nations, it does not provide detailed insights into the tool’s validity for specific subgroups, such as ethnic minorities. Future research should address this gap by examining the psychometric properties of the City BiTS-Short in different population groups. In addition, the psychometric properties of the City BiTS-Short were determined based on data collected using the full version. Thus, future research is needed to evaluate and confirm the specific psychometric qualities of the City BiTS-Short.

## Conclusion

In conclusion, the development of the City BiTS-Short marks a significant advancement in the early identification of traumatic birth and CB-PTSD symptoms. With its strong psychometric properties and broad applicability, this brief tool offers an efficient method for identifying women or their partners who may need further assessment and support. Its integration into a structured two-stage screening process can enhance the early recognition and management of CB-PTSD symptoms, contributing to improved maternal mental health outcomes globally. However, careful consideration of contextual factors, appropriate training for health professionals and ongoing evaluation of effectiveness and implementation are essential to maximise its effectiveness. Further examination of its psychometric properties and validation across diverse populations will strengthen its applicability, ensuring its role in addressing the global burden of traumatic childbirth and its psychological impacts.

## Supplementary material

10.1136/bmjgh-2025-019216 online supplemental appendix 1

10.1136/bmjgh-2025-019216 online supplemental file 1

## Data Availability

Data are available in a public, open access repository.
