# Supplementary material for: A brief international screening tool for traumatic birth and childbirth-related PTSD: the city BiTS-short form
Source: BMJ Glob Health. 2025 Aug 17;10(8):e019216. doi: 10.1136/bmjgh-2025-019216 (PMC12359419; doi:10.1136/bmjgh-2025-019216)
Supplement: online supplemental appendix 1 [file bmjgh-10-8-s001.docx]

**Appendix. City Birth Trauma Scale Short Form**
