# Supplementary material for: A brief international screening tool for traumatic birth and childbirth-related PTSD: the city BiTS-short form
Source: BMJ Glob Health. 2025 Aug 17;10(8):e019216. doi: 10.1136/bmjgh-2025-019216 (PMC12359419; doi:10.1136/bmjgh-2025-019216)
Supplement: online supplemental file 1 [file bmjgh-10-8-s002.pdf]

Supplementary Material
for Ayers *et al.*: Short Form of City BiTS

DBW
daniel.wright@unlv.edu

Abstract

This is the supplementary material statistics materials for Ayers *et al.*'s paper presenting the development of the Short Form of the City BiTS. The data can be accessed through the UK archive. This document is written in **knitr** (Xie, 2015), which combines LaTeX and R. For the value of this approach see Mair (2016). This is written by DBW for use by the other authors who constructed the main paper, but also as a document for others either to replicate these analyses or as a possible way to analyze their own data.

Criteria guiding the approach

The approach taken here is to reduce the number of items in the City BiTs (Ayers, Wrsight, & Thornton, 2018) with six criteria:

1. That there are at most 10 items, but preferably about 6 items or fewer.
2. The items in the short form must cover the four components of intrusions, avoidance, negative mood/cognitions, and hyperarousal. At least one item from each component must be included.
3. That international researchers on the INTERSECT team have not reported (when specifically asked) that any of the items are particularly problematic.
4. That the scoring of the form is simple (e.g., summing values) and can be done with just an individual client (many papers discuss the pros and cons of simply using sumscores, for example, Sijtsma, Ellis, & Borsboom, 2024).
5. The resulting short form should be highly associated with the long form.
6. That the findings for #5 are true for all countries included in the INTERSECT project.

Items Removed

Members of the consortium were contacted and asked if there were items they felt should be removed. During discussion with midwives (and the other groups discussed in the paper) othr issues arose, for example that "jumpy" is awkward to translate. The following items were removed:

B2 Bad dreams or nightmares about the birth (or related to the birth)
B3 Flashbacks to the birth and/or reliving the experience
C2 Trying to avoid things that remind me of the birth (e.g., people, places, TV programs)
D1 Not able to remember details of the birth

D4 Feeling negative about myself or thinking something awful will happen
E1 Feeling irritable or aggressive
E4 Feeling jumpy or easily startled
E5 Problems concentrating
E6 Not sleeping well because of things that are not due to the baby's sleep pattern

Removing C2 presents an issue because there are only two items measuring this component (avoidance) in the long form. This will not affect the running of the exclusive lasso, which does allow having only one item from a component. This means, conceptually, it may be easiest to think of this task as choosing which items to remove than choosing which items to retain.

The following R (R Core Team, 2023) libraries are attached. All must be downloaded first (i.e., those from CRAN using `install.packages`). The citation for these are **xtable** (Dahl, Scott, Roosen, Magnusson, & Swinton, 2019), **lme4** (Bates, Mächler, Bolker, & Walker, 2015), **boot** (Canty & Ripley, 2017), **lattice** (Sarkar, 2008), **plotrix** (Lemon, 2006), **ds4psy** (Neth, 2023), **psych** (Revelle, 2018), **lavaan** (Rosseel, 2012), **flextable** (Gohel & Skintzos, 2024), **e1071** (Meyer, Dimitriadou, Hornik, Weingessel, & Leisch, 2018), **leaps** (Lumley & Miller, 2020), **EFA.dimensions** (O'Connor, 2021), **devtools** (Wickham, Hester, Chang, & Bryan, 2022), **ExclusiveLasso** (Weylandt, Campbell, & Allen, 2018), **DescTools** (Signorell & others, 2018), and **norm** (Schafer, 2023).

```
library(xtable)      # for nicer tables, though not as flexible
                     # as we will probably want, so use tabulate ...
                     # but for paper putting into Word
library(lme4)        # for conditional modes and multilevel models
library(boot)        # inv.logit and bootstrapping
library(lattice)     # for dotplot (rough caterpillar plots)
library(plotrix)     # for draw.ellipse
library(ds4psy)      # for T\"urkiye
library(psych)       # for cor.smooth, alpha, omega
library(lavaan)      # for cfa bifactor
library(flextable)   # may need to remove old systemfonts package
library(e1071)       # for skewness
library(leaps)       # for best subset
library(EFA.dimensions) # for DIMTESTS
#library(devtools)   # need these two lines done once
#install_github("DataSlingers/ExclusiveLasso")
library(ExclusiveLasso)
library(norm)        # for missing; norm2 removed from CRAN
library(DescTools)   # for pseudo-R2 measures
```

The Approaches

Two approaches are considered: the exclusive lasso and the constrained best subset. One difference between these is that the former appears difficult to change to requiring

two items per component. Given the current set–excluding those found troubling by the consortium–component C only has one item any way, this is no longer an issue. The latter approach can be made to be much more flexible, though this is not needed here.

A Brief non-Technical Primer on the Exclusive Lasso

Multiple statistical methods exist for choosing a subset of predictor items in a regression context. Because the predictor variables often correlate, choosing which to include can be complicated (e.g., one that is highly correlated with the outcome variable, may become redundant when others are included). One popular method uses what is called the lasso or the ℓ_1 penalty (Tibshirani, 1996). This is a popular approach in creating a subset of predictors in regression problems and does not have some of the issues of traditional approaches like just removing variables based on the changes in R^2 or other model-fit statistics (e.g., Efron, Hastie, Johnstone, & Tibshirani, 2004). It is both a model selection (which predictors to include) and regularization (prevents some $\widehat{\beta}$ values being too large in magnitude) procedure. Much has been written about this class of procedure and its benefits.

The lasso works by restricting the sum of the absolute values of the standardized $\widehat{\beta}$ estimates to be equal to some value λ (i.e., $\lambda \geq \sum|\widehat{\beta_k}|$, where k denotes the different predictor variables. That the $\widehat{\beta_k}$ are taken to the first power is why it is called ℓ_1. Notations vary; some simple monotonic functions of λ are also used so the notation you see elsewhere may differ. As λ decreases many of the $\widehat{\beta_k}$ become zero, so drop out the model, thus increasing the ease of interpreting the model (i.e., the model selection). The other $\widehat{\beta_k}$ values usually move closer to zero and extremely high (and likely unreliable) values are usually greatly reduced (i.e., the regularization). A predecessor to the lasso is called ridge regression (or ℓ_2 penalty). It works by constraining $\sum\widehat{\beta_k}^2$, which tends to decrease the size of the individual $\widehat{\beta_k}$, but not to zero (squaring the values is why it is called ℓ_2 penalty).

Several extensions to the lasso have been developed for several statistical procedures (for examples, see Hastie, Tibshirani, & Wainwright, 2015). Of interest for satisfying criterion #2 is the exclusive (sometimes elitist, Kowalski, 2009) lasso (Zhou, Jin, & Hoi, 2010). It works by requiring at least one item for each group to be kept (the original lasso ignores group structure). An algorithm for this is described in Campbell and Allen (2017) and implemented in the R package **ExclusiveLasso** R Core Team (2023); Weylandt et al. (2018). Campbell and Allen (2017, their eqn. 1) show this as:

$$\widehat{\beta} = \underset{\beta}{argmin} \; \underbrace{\frac{1}{2}\|y - X\beta\|_2^2}_{\text{ridge penalty}} + \underbrace{\frac{\lambda}{2}\sum_{g\in G}\left(\sum_{i\in G}|\beta_i|\right)^2}_{\text{lasso penalty}} \qquad (1)$$

The key aspect is it applies a ℓ_2 penalty (ridge) between the components, so none of the components drop to zero, and ℓ_1 (lasso) penalty within components so lots drop to zero (and eventually all but one). Other algorithms have recently been proposed (e.g., Meixia Lin & Toh, 2024), but given the popularity and ease of implementation of **ExclusiveLasso** it will be used here.

Caveat: The short form is designed for both researchers, who want a short form to include with a large battery of items, and clinicians, who want a brief screening measure.

For the latter group, it is important that they may quickly calculate a score for an individual. Therefore, taking the sum or mean of items would be reasonable. If the $\widehat{\beta_k}$ values were all equal this would be in some sense statistically optimal also (i.e., the τ-equivalence assumption of Cronbach's α). There is a technique called the fused lasso (Rinaldo, 2009; Tibshirani, Saunders, Rosset, Zhu, & Knight, 2004), which penalizes differences among the $\widehat{\beta_k}$. While this is implemented in the R package **genlasso** (Arnold & Tibshirani, 2016, 2022) it is not used here, nor is there at present a package that implements the two aspects of the exclusive and fused lassos, together. Here, we examine the $\widehat{\beta_k}$ values, which might be used for research purposes, and report the similarity. One aspect of the exclusive lasso is that the estimated β values of the final models tend to be similar.

Constrained Best Subset

Criterion #5, that the short form of a scale is closely associated the long form, could be operationalized as the set that produces the highest R^2 for each number of variables. The least squares estimates of the $\widehat{\beta}$s could be found for all applicable models. Here, based on criterion 4, the values are all the same (so equal to one is convenient). The number of possible models is still quite high. For a k item scale it is 2^k, for example 2^{20} is 1,048,576. Just as the exclusive lasso restricts potential models, here the number of potential models can be reduced if it is required that at least one or two are chosen from each set. Further, models with more than ten variables need not be tested.

This is a flexible approach. Any choice to construct the model, to restrict which models, and to assess fit can be used. The downside is some care is necessary because of R's memory if too many models are estimated and results stored. Procedures could be done within each component to lessen the numbers, but if the number of items is large 2^k becomes to large for our computers. For example, if there were, say 60 items, $2^{60} = 1,152,921,504,606,846,976$, or over 1 quintillion. For comparison, this is about twice the number of seconds since the big bang (assuming contemporary theories of time/the universe). There are many things that can be done to lessen the demand, but here this is not necessary with the smaller number of items after the exclusions.

Here the sum of the potential short form is calculated for each individual model and the correlation between these and the sum for the long form is made.

Analysis

Reading Data

Data were downloaded from the UK Data Service as a tab-delineated file. There is an application process for this. The City BiTs and jurisdiction variable are stored in an object `short`. `-99` is used for missing values for many variables in the downloaded file. These are changed to `NA` for all variables, which is the default for R for missing. An object `items` is created that stores the responses to the B–E items from the BiTs. Further items are removed from these based on input for the consortium. Other variables are added for comparisons discussed towards the end of this report. If replicating this, you would need to change the computer location. Note that if you wish to analyse more variables these can be added to the object `short`. The removal of missing values is done so they are still lined up.

```
dfile <-  paste0("C:\\Users\\wrighd12\\Documents\\",
  "Susan\\Intersect\\intersect_v1_2023.tab")
prev1 <- read.delim(dfile)
short <- prev1[,c(1:6,21:49,10,11,13,20,146,151,153)]
for (i in 1:ncol(short))
  short[short[,i]==-99,i] <- NA
items <- short[,c(9:28,33,34,40,1,2)]
nsxx <- paste0(sort(unique(prev1$jurisdiction))," (n=",
  prettyNum(tapply(prev1$jurisdiction,prev1$jurisdiction,length),
              big.mark=","),")")
```

The exclusive lasso package does not accept any missing values. Responders' data were removed if they were missing 6 or more values from the scale. This is 0.96% so less than 1%.

```
mean(rowSums(is.na(items[,1:20])) < 6)

## [1] 0.9904442

# Removing those with more than 6 missing from scale
items <- items[rowSums(is.na(items[,1:20])) < 6,]
```

This still leaves a small amount ($\approx$ 1%) of missing data. The package **norm** (Schafer, 2023) was used to impute values for these (see that package's manual). There is a **norm2** package, but it is currently not on CRAN, so it may be undergoing some changes. R has many packages for missing data https://cran.r-project.org/web/views/MissingData.html.

```
itemsnomiss <- items[,1:20]
s <- prelim.norm(as.matrix(itemsnomiss))
thetahat <- em.norm(s)

## Iterations of EM:
## 1...2...3...

rngseed(17)
longitems <- items[,1:20] <- imp.norm(s,thetahat,itemsnomiss)
items$sumBiTs <- apply(items[1:20],1,mean)
items <- items[,c(-2,-3,-5,-7,-8,-11,-15,-18,-19,-20)]
```

A variable for the sum of the B–E components from the long form is created and the correlations between each item and this shown below, from highest to lowest. The items corresponding to the highest components are more likely to be of use for the short form, but this approach does not take into account the collinearity of these items, which is high.

```
round(corsxxx <- cor(longitems, items$sumBiTs),2)

##                [,1]
## city_bits_b1 0.66
## city_bits_b2 0.62
## city_bits_b3 0.62
## city_bits_b4 0.70
## city_bits_b5 0.72
## city_bits_c1 0.69
## city_bits_c2 0.64
## city_bits_d1 0.44
## city_bits_d2 0.59
## city_bits_d3 0.72
## city_bits_d4 0.68
## city_bits_d5 0.68
## city_bits_d6 0.67
## city_bits_d7 0.70
## city_bits_e1 0.66
## city_bits_e2 0.58
## city_bits_e3 0.69
## city_bits_e4 0.69
## city_bits_e5 0.60
## city_bits_e6 0.61
```

The correlations range from $r = .44$ to $r = .72$, so are all substantial.

Exclusive Lasso

The subset of items not excluded by the international collaborators will be used to predict the sum score from the long form of City BiTS. The key property of the exclusive lasso is that even if λ increases, there is always at least one item from each group.
The R code to make Figure **??**, is shown below. There is some LaTeX code not shown in the pdf. The first set of code runs the exclusive lasso, mostly with the defaults, or changes as suggested in their manual. λ is varied from .1 to 41.1 in steps of .01. This was based on trial and error to reach the point that the four remaining $\widehat{\beta}$ values were stable.

```
set.seed(3933)
x <- table(substring(sub("city_bits_","",colnames(items[,1:10])),1,1))
groups <- rep(letters[2:5],x)
lseq <- seq(.1, 41.1, .01)
ex1 <- exclusive_lasso(as.matrix(items[,1:10]),
     items$sumBiTs,groups,
        family="gaussian",lambda=lseq,skip_df=TRUE)
```

The following (if #s removed) examines how the changes are occurring. The penultimate line shows with a variable is removed, and then the values are printed.

```
#table(diff(ex1$nnz))
#any(abs(diff(ex1$nnz)) > 1)
droplambda <- diff(ex1$nnz) != 0
round(ex1$coef[,which(droplambda)+1],3)

## 10 x 6 sparse Matrix of class "dgCMatrix"
##
## city_bits_b1 0.073 0.033 0.003 .     .     .
## city_bits_b4 0.093 0.070 0.051 0.049 0.039 0.014
## city_bits_c1 0.138 0.097 0.053 0.047 0.038 0.013
## city_bits_d2 .     .     .     .     .     .
## city_bits_d3 0.030 0.019 0.019 0.019 0.019 0.014
## city_bits_d5 0.055 0.028 0.009 0.006 .     .
## city_bits_d6 0.053 0.021 .     .     .     .
## city_bits_d7 0.054 0.039 0.027 0.025 0.021 .
## city_bits_e2 0.063 .     .     .     .     .
## city_bits_e3 0.107 0.083 0.044 0.039 0.031 0.011
```

Here is the result for just four variables and their variable names.

```
ex1$coef[,max(which(droplambda))+1]

## city_bits_b1 city_bits_b4 city_bits_c1 city_bits_d2 city_bits_d3 city_bits_d5
##   0.00000000   0.01357130   0.01321767   0.00000000   0.01352625   0.00000000
## city_bits_d6 city_bits_d7 city_bits_e2 city_bits_e3
##   0.00000000   0.00000000   0.00000000   0.01069579

(shortformvars <- names(which(ex1$coef[,max(which(droplambda))+1] > 0)))

## [1] "city_bits_b4" "city_bits_c1" "city_bits_d3" "city_bits_e3"
```

They are:

B4 Getting upset when reminded of the birth
C1 Trying to avoid thinking about the birth
D3 Feeling strong negative emotions about the birth (e.g. fear, anger, shame)
E3 Feeling tense and on edge

Figure 1 is made with the following R code (LaTeX code not printed.)

```
par(mar=c(4,6,1,1))
plot(lseq,ex1$coef[1,],col="white",ylab="",las=1,
     xlab=expression(lambda),yaxt='n')
mtext(expression(hat(beta)),2,las=1,3)
axis(2,seq(0,.1,.01),
     sub("0.",".",sprintf("%0.2f",seq(0,.1,.01)),fixed=TRUE),las=1)
for (i in 1:nrow(ex1$coef))
  lines(lseq,ex1$coef[i,],col=as.numeric(as.factor(groups))[i])
```

```
legend("topright",c("re-experiencing","avoidance",
      "negative cognitions and mood","hyperarousal"),col=1:4,lwd=2)
```

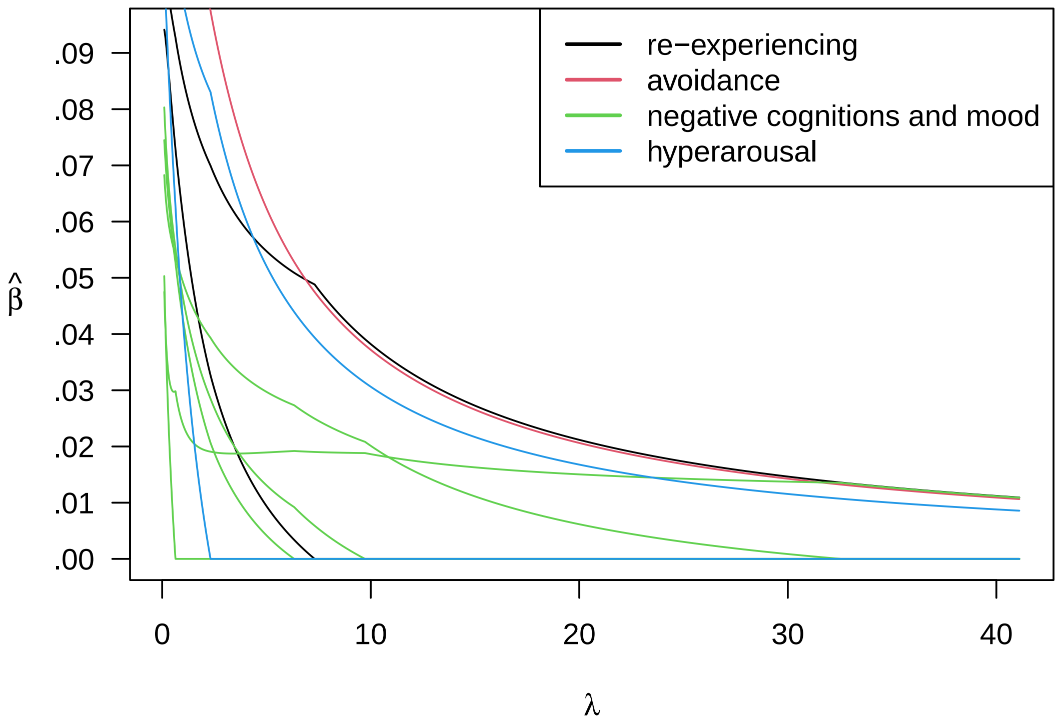

Figure 1

Exclusive lasso as the λ parameter (shrinkage) increases. The exclusive lasso forces the four remaining $\hat{\beta}$s to be similar and non-zero.

The following creates numbers for the table below.

```
not0 <- function(x) sum(x!=0)
numnot0 <- apply(ex1$coef,2,not0)
#which(numnot0 == 10)

attach(as.data.frame(items))
cors <- alphs <- matrix(nrow=7,ncol=4)
tot <- vector(length=7)
for (i in 4:10){
  #print(i)
  ccc <- ex1$coef[,min(which(numnot0 == i))]
  #print(sub("city_bits_","",names(which(ccc != 0))))
  tot[i-3] <- paste(sub("city_bits_","",
                        names(which(ccc != 0))),collapse=" ")
  vnames <- names(which(ccc != 0))
  varlist <- {}
      for (k in 1:i)
        varlist <- cbind(varlist,eval(as.name(vnames[k])))
   corvals <- cor.test(items$sumBiTs,apply(varlist,1,mean,na.rm=TRUE))
   aa <- alpha(varlist,warnings=FALSE,discrete=FALSE)$feldt
   alphaval <- c(aa$alpha$raw_alpha,aa$lower.ci$raw_alpha,
                 aa$upper.ci$raw_alpha)
   cors[i-3,1:4] <- c(i,corvals$estimate,corvals$conf.int)
   alphs[i-3,1:4] <- c(i,alphaval)
           }
```

Table 1 shows these results. This code prints the table to the pdf.

```
corstxt <- matrix(sub("0.",".",
                  sprintf("%0.2f",cors[,2:4]),fixed=TRUE),ncol=3)
alphtxt <- matrix(sub("0.",".",sprintf("%0.2f",alphs[,2:4])),ncol=3)
xtab <- cbind(4:10,tot,corstxt,alphtxt)
colnames(xtab) <- c("\\#vars","variables","$r$","$r_{lb}$","$r_{ub}$",
                    "$\\alpha$","$\\alpha_{lb}$","$\\alpha_{ub}$")
print(xtable(xtab,caption="Best fitting model for each number of variables
      included from the exclusive lasso, showing Pearson's $r$ with the
      sum of original scale and Cronbach's $\\alpha$. The lower and upper
      bounds are shown for the 95\\% confidence intervals.\\vspace{.4cm}",
      label="tab:corexlasso",align="lllcccccc"),
      row.names=FALSE,sanitize.colnames.function=identity,
      caption.placement="top")
```

This code prints the table to a Word file, that then requires a little editing, but not re-typing the numbers, which would introduce a source of possible error. The main manuscript was written in Word.

Table 1

Best fitting model for each number of variables included from the exclusive lasso, showing Pearson's r with the sum of original scale and Cronbach's α. The lower and upper bounds are shown for the 95% confidence intervals.

	#vars	variables	r	r_{lb}	r_{ub}	α	α_{lb}	α_{ub}
1	4	b4 c1 d3 e3	.90	.90	.90	.78	.77	.78
2	5	b4 c1 d3 d7 e3	.93	.93	.93	.80	.80	.81
3	6	b4 c1 d3 d5 d7 e3	.95	.95	.95	.82	.82	.83
4	7	b1 b4 c1 d3 d5 d7 e3	.95	.95	.96	.85	.84	.85
5	8	b1 b4 c1 d3 d5 d6 d7 e3	.96	.96	.96	.86	.86	.87
6	9	b1 b4 c1 d3 d5 d6 d7 e2 e3	.97	.97	.97	.87	.86	.87
7	10	b1 b4 c1 d2 d3 d5 d6 d7 e2 e3	.97	.97	.97	.87	.87	.88

```
fmat <- flextable(as.data.frame(xtab),cwidth=.9)
save_as_docx(fmat,path="xtabshort.docx")
```

The Constrained All Sets Approach

While we examined the output from this approach, the focus for choosing the subset of items was based primarily on the output from the exclusive lasso. This output is included for completeness. These are the groups for the items considered after INTERSECT exclusions:

```
groups

##  [1] "b" "b" "c" "d" "d" "d" "d" "d" "e" "e"
```

This code creates the possible sets of coefficients. Each combination for the 10 items would be $2^{1}0 = 1024$.

```
isgt10 <- function(x) sum(x) > 10
k1 <- ncol(items[,1:10])
x1 <- x2 <- matrix(ncol=k1, nrow=2^k1)
for (i in 0:((2^k1)-1)) {
 coefs <- rev(as.integer(intToBits(i))[1:k1])
 if (isgt10(coefs)) next
 leave <- FALSE
 for (g in groups)
    if (sum(coefs[groups==g]) == 0) leave <- TRUE
 if (leave) next
 x1[i,] <- coefs
#***THIS REQUIRES TWO IN EACH CONSTRUCT
#***Included in case of use to anyone
 # for (g in groups)
#    if (sum(coefs[groups==g]) < 2) leave <- TRUE
# if (leave) next
# x2[i,] <- coefs
 }
#dim(x1)
x1 <- x1[complete.cases(x1),]
#x2 <- x2[complete.cases(x2),]
```

Algorithms are available that to improve the efficiency (i.e., speed) of the search.

```
# this code (changing 2s to 1s) was for the orig at least
# two items runs
cors1 <- cbind(rep(NA,nrow(x1)), rep(NA,nrow(x1)),x1)
cors1[,2] <- apply(x1,1,sum)
qqq <- x1 %*% t(items[,1:10])
corSB <- function(x) cor(items$sumBiTs,x)
cors1[,1] <- apply(qqq,1,corSB)
```

The following creates a plot of all the correlations with the number of variables included, and shows the maximum for each of these.

```
#table(cors1[,2],useNA="always")
#hist(cors1[,1])
#table(is.na(cors1[,1]))
plot(jitter(cors1[,2],factor=.3),cors1[,1],pch=".")
lines(4:10,tapply(cors1[,1],cors1[,2],max),col="red",lwd=2)
```

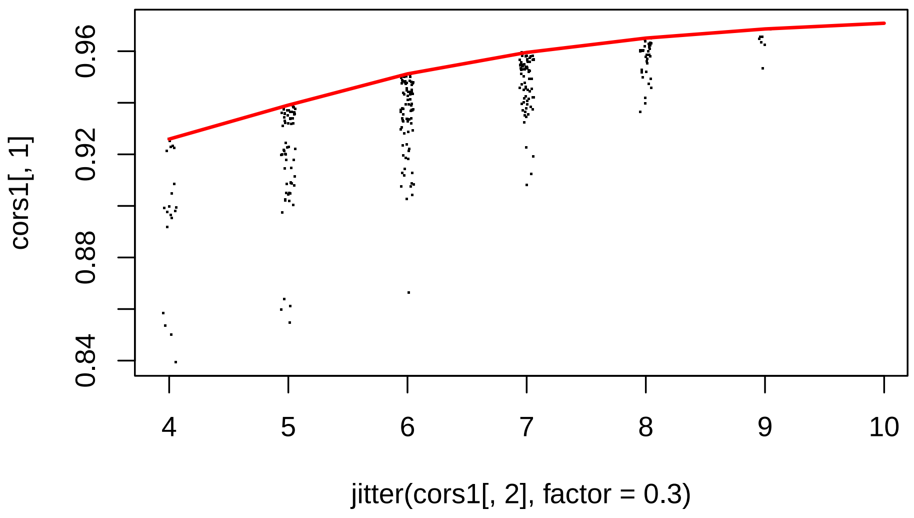


```
tapply(cors1[,1],cors1[,2],max)

##         4         5         6         7         8         9        10
## 0.9259189 0.9390850 0.9512062 0.9595187 0.9650865 0.9686314 0.9708552
```

Chosen Form

The set was based on the exclusive lasso using four items so that each component was represented similarly. Once the form was chosen we checked the association of these with other variables.

```
shortFin <- as.data.frame(cbind(items$city_bits_b4,
                  items$city_bits_c1,
                  items$city_bits_d3,
                  items$city_bits_e3))
colnames(shortFin) <- c("b4","c1","d3","e3")
```

```
shortFinID <- as.data.frame(cbind(
                  id=items$id,
                  b4=items$city_bits_b4,
                  c1=items$city_bits_c1,
                  d3=items$city_bits_d3,
                  e3=items$city_bits_e3))

sumShort <- shortFin$sumShort <- shortFinID$sumShort <-
    apply(shortFin,1,sum)

#cor(shortFin)
#cor(sumShort,items$sumBiTs)
xtabcor <- cor(cbind(shortFin,items$city_bits_g1,
  items$city_bits_g2,items$sumBiTs,items$ptsd_diagnosis),
   use="pairwise.complete.obs")
#pairs(jitter(shortFin,20))
alpha(shortFin,discrete=FALSE)$feldt

##
##    95% confidence boundaries (Feldt)
##  lower alpha upper
##    0.8  0.81  0.81
```

The following prints the correlation table to the pdf. The `threed` function formats the correlations according to APA guidelines, removing the lead zero (since it cannot exceed 1).

```
#change to 3 to match previous
threed <- function(x) sub("0.",".",sprintf("%0.2f",x),fixed=TRUE)
xtabcor <- xtabcor[c(1:4,6,7,5,8,9),c(1:4,6,7,5,8,9)]
xtabcor <- apply(xtabcor,c(1,2),threed)
xtabcor[upper.tri(xtabcor,diag=TRUE)] <- NA
colnames(xtabcor)[5:9] <- rownames(xtabcor)[5:9] <-
  c("g1","g2","SumBiTS","SumShort","PTSDdiag")
xtabcor <- xtabcor[-1,-ncol(xtabcor)]
print(xtable(xtabcor,caption="Correlations among the short form items
       and a few others."),size="small")
```

This prints the table to Word, though editing is required there.

```
xtabcor2 <- cbind(row.names(xtabcor),xtabcor)
fmat <- flextable(as.data.frame(xtabcor2),cwidth=.8)
save_as_docx(fmat,path="xtabcor.docx")
```

	b4	c1	d3	e3	g1	g2	SumBiTS	SumShort
c1	.65							
d3	.64	.62						
e3	.32	.32	.37					
g1	.36	.37	.44	.52				
g2	.30	.31	.37	.45	.67			
SumBiTS	.81	.81	.83	.68	.56	.47		
SumShort	.70	.69	.72	.69	.63	.57	.90	
PTSDdiag	.43	.51	.49	.31	.38	.34	.55	.54

Table 2

Correlations among the short form items and a few others.

Cronbach's α for Short Form and other Psychometric Characteristics with this sample

Creating the table required several lines of code. The `threeCI` function calls the `threed` function, so if using this code you need to have run the previous code first.

```
shortFinX <- shortFin
# removing
shortFinX$jurisdiction <- items$jurisdiction
tabacountries <- matrix(nrow=length(unique(shortFinX$jurisdiction)),
                        ncol=5)
threeCI <- function(x,y){
  citext <- paste0(threed(x), " - ", threed(y))
  return(citext)
  }
for (i in 1:length(unique(shortFinX$jurisdiction))){
  tabacountries[i,1] <- unique(shortFinX$jurisdiction)[i]
  tabacountries[i,4] <-
     threed(as.numeric(alpha(shortFinX[shortFinX[,6]==
            unique(shortFinX$jurisdiction)[i],1:4])$feldt$alpha))
  tabacountries[i,5] <-
      paste0(threed(as.numeric(alpha(shortFinX[shortFinX[,6]==
            unique(shortFinX$jurisdiction)[i],1:4])$feldt$lower.ci)), " - ",
     threed(as.numeric(alpha(shortFinX[shortFinX[,6]==
            unique(shortFinX$jurisdiction)[i],1:4])$feldt$upper.ci)))
  tabacountries[i,2] <-
      threed(as.numeric(cor.test(
          shortFinX$sumShort[shortFinX[,6]==unique(shortFinX$jurisdiction)[i]],
          items$sumBiTs[shortFinX[,6]==
                          unique(shortFinX$jurisdiction)[i]])$estimate))
  tabacountries[i,3] <- paste0(
      threed(as.numeric(cor.test(
          shortFinX$sumShort[shortFinX[,6]==unique(shortFinX$jurisdiction)[i]],
          items$sumBiTs[shortFinX[,6]==
                    unique(shortFinX$jurisdiction)[i]])$conf.int[1])), " - ",
      threed(as.numeric(cor.test(
          shortFinX$sumShort[shortFinX[,6]==unique(shortFinX$jurisdiction)[i]],
          items$sumBiTs[shortFinX[,6]==
                          unique(shortFinX$jurisdiction)[i]])$conf.int[2])))
  }
```

This prints the table to pdf.

```
colnames(tabacountries) <- c("Country","$r$","$r~95\\%~CI$","$\\alpha$",
                             "$\\alpha~95\\%~CI$")
tabacountries[,1] <- nsxx
#
#  paste0(tabacountries[,1]," (n= ",
#      prettyNum(tapply(shortFinX$jurisdiction,shortFinX$jurisdiction,length),
#                 big.mark=","),")")
```

```
tabacountries <- tabacountries[order(tabacountries[,1]),]
print(xtable(tabacountries,
      caption="The $r$ between the sum of the four-item
      short form and Cronbach $\\alpha$ values for the short form,
      for all countries.\\vspace{.4cm}",
      label="tab:bycountry",align="llcccc"),
      row.names=FALSE,sanitize.colnames.function=identity,
      caption.placement="top")
```

This prints the table to Word, and then requires a bit of editing.

```
fmat <- flextable(as.data.frame(tabacountries),cwidth=.9)
save_as_docx(fmat,path="tabacountriesshort.docx")
```

Table 3

The r between the sum of the four-item short form and Cronbach α values for the short form, for all countries.

	Country	r	r 95% CI	α	α 95% CI
1	Australia (n=166)	.84	.82 - .85	.62	.59 - .65
2	Brazil (n=596)	.87	.83 - .90	.70	.64 - .76
3	Chile (n=127)	.86	.83 - .89	.68	.62 - .74
4	Croatia (n=380)	.89	.86 - .91	.73	.67 - .78
5	Cyprus (n=142)	.86	.83 - .88	.67	.60 - .72
6	Czechia (n=246)	.86	.83 - .88	.71	.66 - .75
7	Estonia (n=285)	.94	.93 - .95	.86	.83 - .88
8	Germany (n=1,644)	.85	.82 - .88	.77	.72 - .81
9	Iceland (n=701)	.87	.84 - .90	.76	.70 - .80
10	Ireland (n=274)	.89	.87 - .91	.70	.65 - .74
11	Israel (n=248)	.86	.83 - .89	.67	.60 - .73
12	Italy (n=211)	.87	.84 - .89	.74	.70 - .78
13	Lithuania (n=328)	.86	.84 - .88	.75	.72 - .78
14	Malawi (n=248)	.86	.81 - .90	.69	.59 - .77
15	Nepal (n=490)	.84	.81 - .87	.68	.62 - .73
16	Nigeria (n=406)	.86	.80 - .89	.64	.53 - .73
17	Norway (n=221)	.88	.86 - .89	.69	.65 - .72
18	Pakistan (n=335)	.89	.86 - .91	.76	.71 - .80
19	Poland (n=296)	.84	.79 - .88	.70	.62 - .76
20	Portugal (n=227)	.95	.94 - .96	.82	.78 - .85
21	Romania (n=135)	.95	.94 - .96	.87	.85 - .89
22	Saudi Arabia (n=248)	.83	.76 - .87	.69	.60 - .77
23	Serbia (n=267)	.97	.96 - .98	.95	.94 - .96
24	Slovakia (n=437)	.83	.79 - .87	.53	.42 - .62
25	Slovenia (n=236)	.85	.80 - .89	.69	.61 - .76
26	Spain (n=254)	.90	.89 - .91	.77	.75 - .79
27	Sweden (n=469)	.89	.85 - .91	.76	.71 - .81
28	Switzerland (n=247)	.88	.85 - .91	.76	.71 - .81
29	Turkiye (n=1,013)	.88	.84 - .91	.64	.55 - .73
30	UAE (n=165)	.84	.80 - .88	.68	.60 - .74
31	UK (n=260)	.90	.87 - .92	.79	.74 - .83

Validity

This section is just printing the output. Tables are made elsewhere.

```
valx <- merge(shortFinID,short,by.x="id")
# Depression
with(valx,table(epds_score,useNA="always"))

## epds_score
##    0    1    2    3    4    5    6    7    8    9   10   11   12   13   14   15
## 1246  641  843  789  796  677  655  632  551  507  421  383  363  319  289  250
##   16   17   18   19   20   21   22   23   24   25   26   27   28   29   30 <NA>
##  251  236  199  234  215  302  104   50   30   20   15   19   27    4    4  122

with(valx,table(epds13_high_spec,useNA="always"))

## epds13_high_spec
##    0    1 <NA>
## 8504 2568  122

#Birth distress
with(valx,table(birth_trauma_perception,useNA="always"))

## birth_trauma_perception
##    0    1    2    3    4    5    6    7    8    9   10 <NA>
## 3153 1001  997  917  715 1519  538  721  651  269  664   49

#Birth complications
with(valx,table(mother_complications,useNA="always"))

## mother_complications
##    0    1    2 <NA>
## 6883 3424  877   10

with(valx,table(infant_complications,useNA="always"))

## infant_complications
##    0    1    2 <NA>
## 9036 1643  497   18

#Birth method
with(valx,table(birth_method,useNA="always"))

## birth_method
##    1    2    3    4 <NA>
## 7188  689 1754 1547   16
```

```
cor1 <- with(valx,
   cor.test(epds_score,sumShort,use="pairwise.complete.obs"))
cor2 <- with(valx,
   cor.test(birth_trauma_perception,sumShort,use="pairwise.complete.obs"))
```

There are a few extra missing for these. Pairwise complete observation methods are used.

The correlation between the short form score and EPDS score was $r = .42, p < .001, n = 11,070, CI = (.40, .40)$. The correlation between the short form score and birth trauma perception was $r = .50, p < .001, n = 11,143, CI = (.49, .49)$.

```
library(lsr) #for ciMean
library(effectsize)
with(valx,tapply(sumShort,epds13_high_spec,mean,na.rm=TRUE))

##        0        1
## 1.611312 3.849371

with(valx,tapply(sumShort,epds13_high_spec,ciMean,na.rm=TRUE))

## $`0`
##          2.5%    97.5%
## [1,] 1.564823 1.657802
##
## $`1`
##          2.5%    97.5%
## [1,] 3.715464 3.983278

with(valx,t.test(sumShort~epds13_high_spec))

##
##  Welch Two Sample t-test
##
## data:  sumShort by epds13_high_spec
## t = -30.96, df = 3209.5, p-value < 2.2e-16
## alternative hypothesis: true difference in means between group 0 and group 1 is not equa
## 95 percent confidence interval:
##  -2.379798 -2.096320
## sample estimates:
## mean in group 0 mean in group 1
##        1.611312        3.849371

with(valx,cohens_d(sumShort~epds13_high_spec))

## Cohen's d |         95% CI
## --------------------------
```

```
## -0.88     | [-0.93, -0.84]
##
## - Estimated using pooled SD.
```

```
#Birth complications
with(valx,tapply(sumShort,mother_complications,mean,na.rm=TRUE))
```

```
##        0        1        2
## 1.713085 2.528821 3.741059
```

```
with(valx,tapply(sumShort,mother_complications,ciMean,na.rm=TRUE))
```

```
## $`0`
##          2.5%    97.5%
## [1,] 1.657425 1.768746
##
## $`1`
##          2.5%    97.5%
## [1,] 2.432397 2.625246
##
## $`2`
##          2.5%    97.5%
## [1,] 3.504266 3.977852
```

```
with(valx,oneway.test(sumShort~mother_complications))
```

```
##
##  One-way analysis of means (not assuming equal variances)
##
## data:  sumShort and mother_complications
## F = 212.84, num df = 2.0, denom df = 2163.8, p-value < 2.2e-16
```

```
with(valx,eta_squared(oneway.test(sumShort~mother_complications))$Eta2)
```

```
## [1] 0.1643884
```

```
with(valx,tapply(sumShort,infant_complications,mean,na.rm=TRUE))
```

```
##        0        1        2
## 1.893033 2.789602 4.106640
```

```
with(valx,tapply(sumShort,infant_complications,ciMean,na.rm=TRUE))
```

```
## $`0`
##          2.5%    97.5%
## [1,] 1.841744 1.944322
##
```

```
## $`1`
##          2.5%    97.5%
## [1,] 2.640038 2.939166
##
## $`2`
##          2.5%    97.5%
## [1,] 3.775051 4.438229

with(valx,oneway.test(sumShort~infant_complications))

##
##  One-way analysis of means (not assuming equal variances)
##
## data:  sumShort and infant_complications
## F = 138.93, num df = 2.0, denom df = 1100.2, p-value < 2.2e-16

with(valx,eta_squared(oneway.test(sumShort~infant_complications))$Eta2)

## [1] 0.2016295

#Birth method
with(valx,tapply(sumShort,birth_method,mean,na.rm=TRUE))

##        1        2        3        4
## 1.781044 2.857773 3.298678 2.060589

with(valx,tapply(sumShort,birth_method,ciMean,na.rm=TRUE))

## $`1`
##         2.5%    97.5%
## [1,] 1.72543 1.836657
##
## $`2`
##          2.5%    97.5%
## [1,] 2.625395 3.090151
##
## $`3`
##          2.5%    97.5%
## [1,] 3.143527 3.453829
##
## $`4`
##          2.5%    97.5%
## [1,] 1.929505 2.191674

with(valx,oneway.test(sumShort~birth_method))
```

```
##
##  One-way analysis of means (not assuming equal variances)
##
## data:  sumShort and birth_method
## F = 127.38, num df = 3.0, denom df = 2174.5, p-value < 2.2e-16
```

```
with(valx,eta_squared(oneway.test(sumShort~birth_method))$Eta2)
```

```
## [1] 0.1494669
```

The other variables appear categorical.

```
tabacountries2 <- matrix(nrow=length(unique(shortFinX$jurisdiction)),
                         ncol=9)
tabacountries2[,1:5] <- tabacountries

for (i in 1:length(unique(valx$jurisdiction))){
  tabacountries2[i,1] <- unique(valx$jurisdiction)[i]
  tabacountries2[i,6] <-
      threed(as.numeric(cor.test(
          valx$sumShort[valx[,7]==unique(valx$jurisdiction)[i]],
          valx$epds_score[valx[,7]==
                        unique(valx$jurisdiction)[i]])$estimate))
  tabacountries2[i,7] <- paste0(
      threed(as.numeric(cor.test(
          valx$sumShort[valx[,7]==unique(valx$jurisdiction)[i]],
          valx$epds_score[valx[,7]==
            unique(valx$jurisdiction)[i]])$conf.int[1])), " - ",
      threed(as.numeric(cor.test(
          valx$sumShort[valx[,7]==unique(valx$jurisdiction)[i]],
          valx$epds_score[valx[,7]==
                    unique(valx$jurisdiction)[i]])$conf.int[2])))
  tabacountries2[i,8] <-
      threed(as.numeric(cor.test(
          valx$sumShort[valx[,7]==unique(valx$jurisdiction)[i]],
          valx$birth_trauma_perception[valx[,7]==
                       unique(valx$jurisdiction)[i]])$estimate))
  tabacountries2[i,9] <- paste0(
      threed(as.numeric(cor.test(
          valx$sumShort[valx[,7]==unique(valx$jurisdiction)[i]],
          valx$birth_trauma_perception[valx[,7]==
                unique(valx$jurisdiction)[i]])$conf.int[1])), " - ",
      threed(as.numeric(cor.test(
          valx$sumShort[valx[,7]==unique(valx$jurisdiction)[i]],
          valx$birth_trauma_perception[valx[,7]==
                unique(valx$jurisdiction)[i]])$conf.int[2])))
  }
```

```r
colnames(tabacountries2) <- c("Country","$r$","$r~95\\%~CI$",
            "$\\alpha$","$\\alpha~95\\%~CI$",
            "depr","deprCI","perctrauma","percI")
tabacountries2 <- tabacountries2[order(tabacountries2[,1]),]
print(xtable(tabacountries2,
      caption="The $r$ between the sum of the four-item
      short form and Cronbach $\\alpha$ values for the short form,
      for all countries. This was for the co-authors to look at and is
      not used in the paper.\\vspace{.4cm}",
      label="tab:bycountry2",align="llcccccccc"),
      row.names=FALSE,sanitize.colnames.function=identity,
      caption.placement="top",size="footnotesize")
```

Table 4

The r between the sum of the four-item short form and Cronbach α values for the short form, for all countries. This was for the co-authors to look at and is not used in the paper.

	Country	r	r 95% *CI*	α	α 95% *CI*	depr	deprCI	perctrauma	percI
1	Australia	.84	.82 - .85	.62	.59 - .65	.53	.41 - .63	.52	.40 - .62
2	Brazil	.87	.83 - .90	.70	.64 - .76	-.42	-.49 - -.36	.48	.42 - .54
3	Chile	.86	.83 - .89	.68	.62 - .74	.54	.41 - .66	.41	.25 - .55
4	Croatia	.89	.86 - .91	.73	.67 - .78	-.34	-.43 - -.25	.54	.46 - .60
5	Cyprus	.86	.83 - .88	.67	.60 - .72	.49	.35 - .60	.52	.38 - .63
6	Czechia	.86	.83 - .88	.71	.66 - .75	.49	.39 - .58	.45	.34 - .54
7	Estonia	.94	.93 - .95	.86	.83 - .88	.50	.41 - .58	.48	.39 - .57
8	Germany	.85	.82 - .88	.77	.72 - .81	.45	.41 - .48	.43	.39 - .47
9	Iceland	.87	.84 - .90	.76	.70 - .80	.56	.51 - .61	.54	.48 - .59
10	Ireland	.89	.87 - .91	.70	.65 - .74	.59	.50 - .66	.60	.52 - .67
11	Israel	.86	.83 - .89	.67	.60 - .73	.46	.35 - .55	.46	.36 - .55
12	Italy	.87	.84 - .89	.74	.70 - .78	.41	.29 - .52	.53	.42 - .62
13	Lithuania	.86	.84 - .88	.75	.72 - .78	.57	.49 - .64	.54	.45 - .61
14	Malawi	.86	.81 - .90	.69	.59 - .77	.64	.56 - .71	.79	.74 - .83
15	Nepal	.84	.81 - .87	.68	.62 - .73	.67	.62 - .71	.27	.19 - .35
16	Nigeria	.86	.80 - .89	.64	.53 - .73	.43	.35 - .50	.25	.16 - .34
17	Norway	.84	.80 - .88	.68	.60 - .74	.59	.49 - .67	.67	.59 - .74
18	Pakistan	.88	.86 - .89	.69	.65 - .72	.72	.66 - .76	.35	.26 - .44
19	Poland	.89	.86 - .91	.76	.71 - .80	.49	.40 - .58	.59	.51 - .66
20	Portugal	.84	.79 - .88	.70	.62 - .76	.43	.31 - .53	.52	.41 - .61
21	Romania	.95	.94 - .96	.82	.78 - .85	.43	.28 - .55	.46	.32 - .59
22	Saudi Arabia	.95	.94 - .96	.87	.85 - .89	.76	.70 - .81	.70	.63 - .76
23	Serbia	.83	.76 - .87	.69	.60 - .77	.47	.37 - .56	.46	.36 - .55
24	Slovakia	.97	.96 - .98	.95	.94 - .96	.51	.44 - .58	.58	.52 - .64
25	Slovenia	.88	.84 - .91	.64	.55 - .73	.52	.41 - .62	.58	.48 - .66
26	Spain	.83	.79 - .87	.53	.42 - .62	.50	.40 - .59	.41	.30 - .51
27	Sweden	.85	.80 - .89	.69	.61 - .76	.59	.53 - .65	.58	.51 - .64
28	Switzerland	.90	.89 - .91	.77	.75 - .79	.49	.39 - .58	.47	.37 - .57
29	Turkiye	.88	.85 - .91	.76	.71 - .81	.53	.49 - .57	.68	.64 - .71
30	UAE	.89	.85 - .91	.76	.71 - .81	.50	.37 - .61	.13	-.02 - .28
31	UK	.90	.87 - .92	.79	.74 - .83	.49	.39 - .58	.62	.54 - .69

```
fmat <- flextable(as.data.frame(tabacountries2),cwidth=.9)
save_as_docx(fmat,path="tabacountriesshort2.docx")
```

Screening Measure

With a short form, many users will use the results to start a conversation with the mother. Certain values may prompt the user to suggest using the long form or even to make a referral (though we recommend using the long form to make referrals as this will provide more information for future appointments).

```
table(round(sumShort)) # since imputation of missing means not

##
##    0    1    2    3    4    5    6    7    8    9   10   11   12
## 4345 1842 1581 1030  579  416  348  279  302  167  131   88   86

                       # all integers
(perc0 <- round(100*mean(round(sumShort)==0)))

## [1] 39
```

```
hist(sumShort,xlab="City BiTS-Short total score",main="",
     xaxt='n')
axis(1,seq(0,12,2)-.5,seq(0,12,2))
text(1.8,3400,"39% at zero")
```

One approach is looking to see if there is a gap in the histogram. From Figure 2 there does not appear to be any. An alternative is examining how sum scores for the short form are related to the PTSD diagnosis variable Veall and Zimmermann (1992) say the McKelvey and Zavoina measure offers a good comparison to standard R^2 from linear OLS models. Its size, here, is fairly large given only four variables are used, and simply summed together.

```
logreg <- with(valx,glm(ptsd_diagnosis ~ sumShort,family="binomial"))
summary(logreg)

##
## Call:
## glm(formula = ptsd_diagnosis ~ sumShort, family = "binomial")
##
## Coefficients:
##             Estimate Std. Error z value Pr(>|z|)
## (Intercept) -5.49268    0.11773  -46.65   <2e-16 ***
## sumShort     0.63168    0.01698   37.20   <2e-16 ***
```

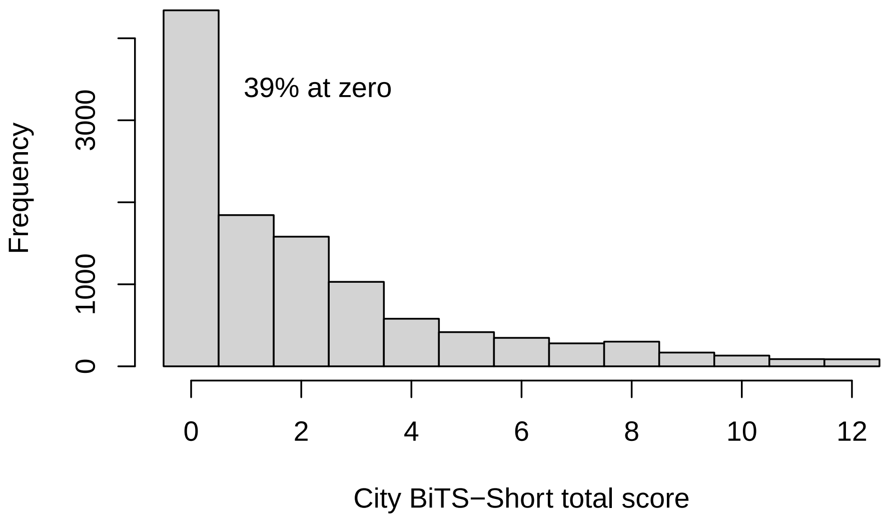

Figure 2

Histogram of the summary of the short form responses.

```
## ---
## Signif. codes:  0 '***' 0.001 '**' 0.01 '*' 0.05 '.' 0.1 ' ' 1
##
## (Dispersion parameter for binomial family taken to be 1)
##
##     Null deviance: 5303.8  on 11174  degrees of freedom
## Residual deviance: 2906.7  on 11173  degrees of freedom
##   (19 observations deleted due to missingness)
## AIC: 2910.7
##
## Number of Fisher Scoring iterations: 7

PseudoR2(logreg,which="McKelveyZavoina")

## McKelveyZavoina
##       0.4679509
```

Figure 3

```
preds <- predict(logreg,type="response",newdata=data.frame(sumShort=0:12))
plot(0:12,preds,type="b",las=1,xlab="City BiTS-Short total score",
     ylab="Prob. PTSD diagnosis",ylim=c(0,1))
```

```
abline(h=.25)
abline(v=7)
```

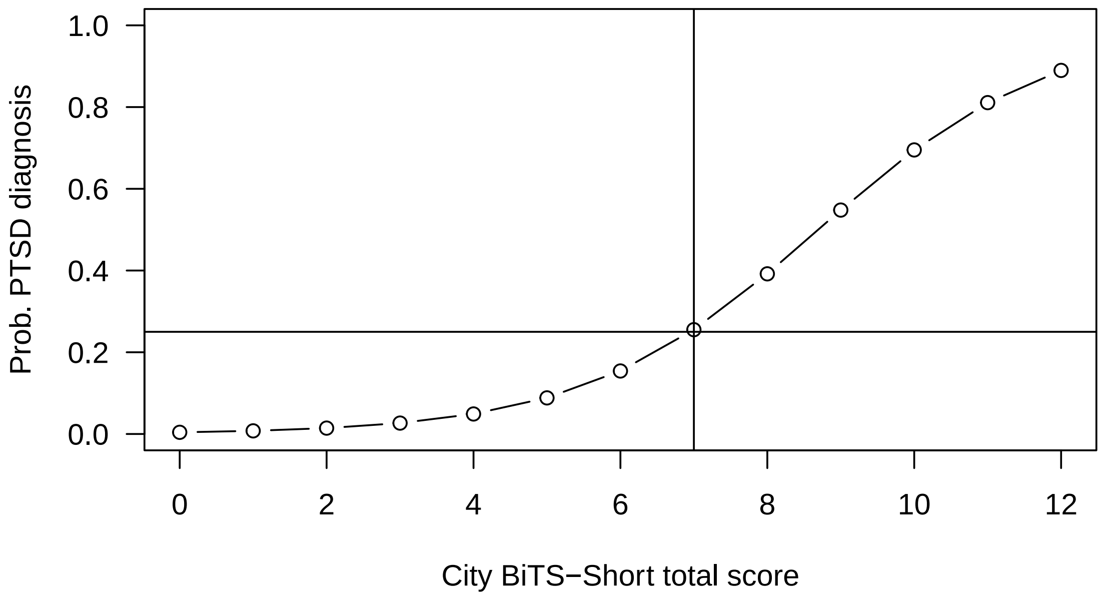

Figure 3

Predicted probability of saying yes to the PTSD variable from the sum of the short form responses.

```
cbind(0:12,preds)

##              preds
## 1   0 0.004099905
## 2   1 0.007683259
## 3   2 0.014353359
## 4   3 0.026658441
## 5   4 0.048988315
## 6   5 0.088325041
## 7   6 0.154129231
## 8   7 0.255233772
## 9   8 0.391930221
## 10  9 0.547971699
## 11 10 0.695119427
```

```
## 12 11 0.810896848
## 13 12 0.889685523
```

References

Arnold, T. B., & Tibshirani, R. J. (2016). Efficient implementations of the generalized lasso dual path algorithm. *Journal of Computational and Graphical Statistics*, *25*(1), 1-27. doi: 10.1080/10618600.2015.1008638

Arnold, T. B., & Tibshirani, R. J. (2022). **genlasso**: Path algorithm for generalized lasso problems [Computer software manual]. Retrieved from `https://CRAN.R-project.org/package=genlasso` (R package version 1.6.1)

Ayers, S., Wrsight, D. B., & Thornton, A. (2018). Development of a measure of postpartum PTSD: The City Birth Trauma Scale. *Frontiers in Psychiatry*, *9*, 409. doi: 10.3389/fpsyt.2018.00409

Bates, D., Mächler, M., Bolker, B., & Walker, S. (2015). Fitting linear mixed-effects models using **lme4**. *Journal of Statistical Software*, *67*(1), 1-48. doi: 10.18637/jss.v067.i01

Campbell, F., & Allen, G. I. (2017). Within group variable selection through the exclusive lasso. *Electronic Journal of Statistics*, *11*(2), 4220 - 4257. doi: 10.1214/17-EJS1317

Canty, A., & Ripley, B. D. (2017). **boot**: Bootstrap R (S-Plus) functions [Computer software manual]. (R package version 1.3-20)

Dahl, D. B., Scott, D., Roosen, C., Magnusson, A., & Swinton, J. (2019). **xtable**: Export tables to LaTeX or HTML [Computer software manual]. Retrieved from `https://CRAN.R-project.org/package=xtable` (R package version 1.8-4)

Efron, B., Hastie, T., Johnstone, I., & Tibshirani, R. (2004). Least angle regression. *The Annals of Statistics*(2), 407-499. doi: 10.1214/009053604000000067

Gohel, D., & Skintzos, P. (2024). **flextable**: Functions for tabular reporting [Computer software manual]. Retrieved from `https://CRAN.R-project.org/package=flextable` (R package version 0.9.6)

Hastie, T., Tibshirani, R., & Wainwright, M. (2015). *Statistical learning with sparsity: The lasso and generalizations.* Boca Raton, FL: CRC Press.

Kowalski, M. (2009). Sparse regression using mixed norms. *Applied and Computational Harmonic Analysis*, *27*(3), 303-324. doi: 10.1016/j.acha.2009.05.006

Lemon, J. (2006). **plotrix**: A package in the red light district of R. *R-News*, *6*(4), 8-12.

Lumley, T., & Miller, A. (2020). **leaps**: Regression subset selection [Computer software manual]. Retrieved from `https://CRAN.R-project.org/package=leaps` (R package version 3.1)

Mair, P. (2016). Thou shalt be reproducible! a technology perspective. *Frontiers in Psychology*, *7*. doi: 10.3389/fpsyg.2016.01079

Meixia Lin, D. S., Yancheng Yuan, & Toh, K.-C. (2024). A highly efficient algorithm for solving exclusive lasso problems. *Optimization Methods and Software*, ****(**), 1-30. doi: 10.1080/10556788.2023.2253356

Meyer, D., Dimitriadou, E., Hornik, K., Weingessel, A., & Leisch, F. (2018). **e1071**: Misc functions of the Department of Statistics, Probability Theory Group (formerly:

E1071), TU Wien [Computer software manual]. Retrieved from https://CRAN.R-project.org/package=e1071 (R package version 1.7-0)

Neth, H. (2023). **ds4psy**: Data science for psychologists [Computer software manual]. Konstanz, Germany. Retrieved from https://CRAN.R-project.org/package=ds4psy (R package (version 1.0.0, September 15, 2023); Textbook at <https://bookdown.org/hneth/ds4psy/>.) doi: 10.5281/zenodo.7229812

O'Connor, B. P. (2021). **EFA.dimensions**: Exploratory factor analysis functions for assessing dimensionality [Computer software manual]. Retrieved from https://CRAN.R-project.org/package=EFA.dimensions (R package version 0.1.7.2)

R Core Team. (2023). R: A language and environment for statistical computing [Computer software manual]. Vienna, Austria. Retrieved from https://www.R-project.org/

Revelle, W. (2018). **psych**: Procedures for psychological, psychometric, and personality research [Computer software manual]. Evanston, Illinois. Retrieved from https://CRAN.R-project.org/package=psych (R package version 1.8.10)

Rinaldo, A. (2009). Properties and refinements of the fused lasso. *The Annals of Statistics*, *37*(5B), 2922-2952.

Rosseel, Y. (2012). **lavaan**: An R package for structural equation modeling. *Journal of Statistical Software*, *48*(2), 1-36. Retrieved from http://www.jstatsoft.org/v48/i02/

Sarkar, D. (2008). ***Lattice**: Multivariate data visualization with R*. New York: Springer. Retrieved from http://lmdvr.r-forge.r-project.org (ISBN 978-0-387-75968-5)

Schafer, J. L. (2023). **norm**: Analysis of multivariate normal datasets with missing values [Computer software manual]. Retrieved from https://CRAN.R-project.org/package=norm (R package version 1.0-11.1)

Signorell, A., & others. (2018). **DescTools**: Tools for descriptive statistics [Computer software manual]. Retrieved from https://cran.r-project.org/package=DescTools (R package version 0.99.24)

Sijtsma, K., Ellis, J. L., & Borsboom, D. (2024). Recognize the value of the sum score, psychometrics' greatest accomplishment. *Psychometrika*, *89*, 84-117. doi: 10.1007/s11336-024-09964-7

Tibshirani, R. (1996). Regression shrinkage and selection via the lasso. *Journal of the Royal Statistical Society: Series B (Methodological)*, *58*, 267-288. doi: 10.1111/j.2517-6161.1996.tb02080.x

Tibshirani, R., Saunders, M., Rosset, S., Zhu, J., & Knight, K. (2004). Sparsity and smoothness via the fused lasso. *Journal of the Royal Statistical Society: Series B*, *67*(1), 91-108. doi: 10.1111/j.1467-9868.2005.00490.x

Veall, M. R., & Zimmermann, K. F. (1992). Evalutating pseudo-r^2's for binary probit models. *Quality & Quantity*, *28*, 151-164.

Weylandt, M., Campbell, F., & Allen, G. (2018). **ExclusiveLasso**: Generalized linear models with the exclusive lasso penalty [Computer software manual]. Retrieved from https://github.com/DataSlingers/ExclusiveLasso (R package version 0.0)

Wickham, H., Hester, J., Chang, W., & Bryan, J. (2022). **devtools**: Tools to make developing r packages easier [Computer software manual]. Retrieved from https://CRAN.R-project.org/package=devtools (R package version 2.4.5)

Xie, Y. (2015). *Dynamic documents with R and **knitr*** (2nd ed.). Boca Raton, FL: Chapman

and Hall/CRC.

Zhou, Y., Jin, R., & Hoi, S. C. (2010). Exclusive lasso for multi-task feature selection. In Y. W. Teh & M. Titterington (Eds.), *Proceedings of the thirteenth international conference on artificial intelligence and statistics* (Vol. 9, p. 988-995). Chia Laguna Resort, Sardinia, Italy: PMLR.
